# Observations on the Physiological and Therapeutical Effects of Galvanization of the Sympathetic

**Published:** 1871-06

**Authors:** 


PAMPHLETS RECEIVED.

Observations on the Physiological and Therapeutical Effects of Galvanization of the Sympathetic. By A. D. Rockwell, A.M., M.D., and Geo. M. Beard, A.M., M.D. New York Printing Co., 81–83 Centre Street.

